# TFutils: Data structures for transcription factor bioinformatics

**DOI:** 10.12688/f1000research.17976.2

**Published:** 2019-05-17

**Authors:** Benjamin J. Stubbs, Shweta Gopaulakrishnan, Kimberly Glass, Nathalie Pochet, Celine Everaert, Benjamin Raby, Vincent Carey

**Affiliations:** 1 Channing Division of Network Medicine, Brigham and Women's Hospital, Harvard Medical School, Boston, MA, 02115, USA; 2 Broad Institute, Cambridge, MA, 02142, USA; 3 Department of Neurology, Brigham and Women's Hospital, Harvard Medical School, Boston, MA, 02115, USA; 4 Pulmonary Genetics Center, Children's Hospital Boston, Boston, MA, 02115, USA

**Keywords:** Transcription factors, Gene expression, Gene regulation, Bioconductor

## Abstract

DNA transcription is intrinsically complex. Bioinformatic work with transcription factors (TFs) is complicated by a multiplicity of data resources and annotations. The Bioconductor package TFutils includes data structures and functions to enhance the precision and utility of integrative analyses that have components involving TFs. TFutils provides catalogs of human TFs from three reference sources (CISBP, HOCOMOCO, and GO), a catalog of TF targets derived from MSigDb, and multiple approaches to enumerating TF binding sites, including an interface to results of 690 ENCODE experiments. Aspects of integration of TF binding patterns and genome-wide association study results are explored in examples.

## Introduction

A central concern of genome biology is improving understanding of gene transcription. In simple terms, transcription factors (TFs) are proteins that bind to DNA, typically near gene promoter regions. The role of TFs in gene expression variation is of great interest. Progress in deciphering genetic and epigenetic processes that affect TF abundance and function will be essential in clarifying and interpreting gene expression variation patterns and their effects on phenotype. Difficulties of identifying functional binding of TFs, and opportunities for using information of TF binding in systems biology contexts, are reviewed in Lambert *et al*. ^1^ and Weirauch *et al*. ^2^.

This paper describes an R/Bioconductor package called TFutils, which assembles various resources intended to clarify and unify approaches to working with TF concepts in bioinformatic analysis. Computations described in this paper can be carried out with Bioconductor version 3.8. The package can be installed with


# use install.packages("BiocManager") if not already available
library(BiocManager)
install("TFutils")


In the next section we describe the basic concepts of enumerating and classifying TFs, enumerating TF targets, and representing genome-wide quantification of TF binding affinity. This is followed by a review of the key data structures and functions provided in the package, and an example in cancer informatics.

The present paper does not deal directly with the manipulation or interpretation of sequence motifs. An excellent Bioconductor package that synthesizes many approaches to these tasks is *universalmotif*.

A complete reference manual enumerating all functions and data sets in the package is available at: http://bioconductor.org/packages/release/bioc/manuals/TFutils/man/TFutils.pdf

## Basic concepts of transcription factor bioinformatics

### Enumerating transcription factors

Given the importance of the topic, it is not surprising that a number of bioinformatic research groups have published catalogs of transcription factors along with metadata about their features. Standard nomenclature for TFs has yet to be established. Gene symbols, motif sequences, and position-weight matrix catalog entries have all been used as TF identifiers.

**Figure 1.**
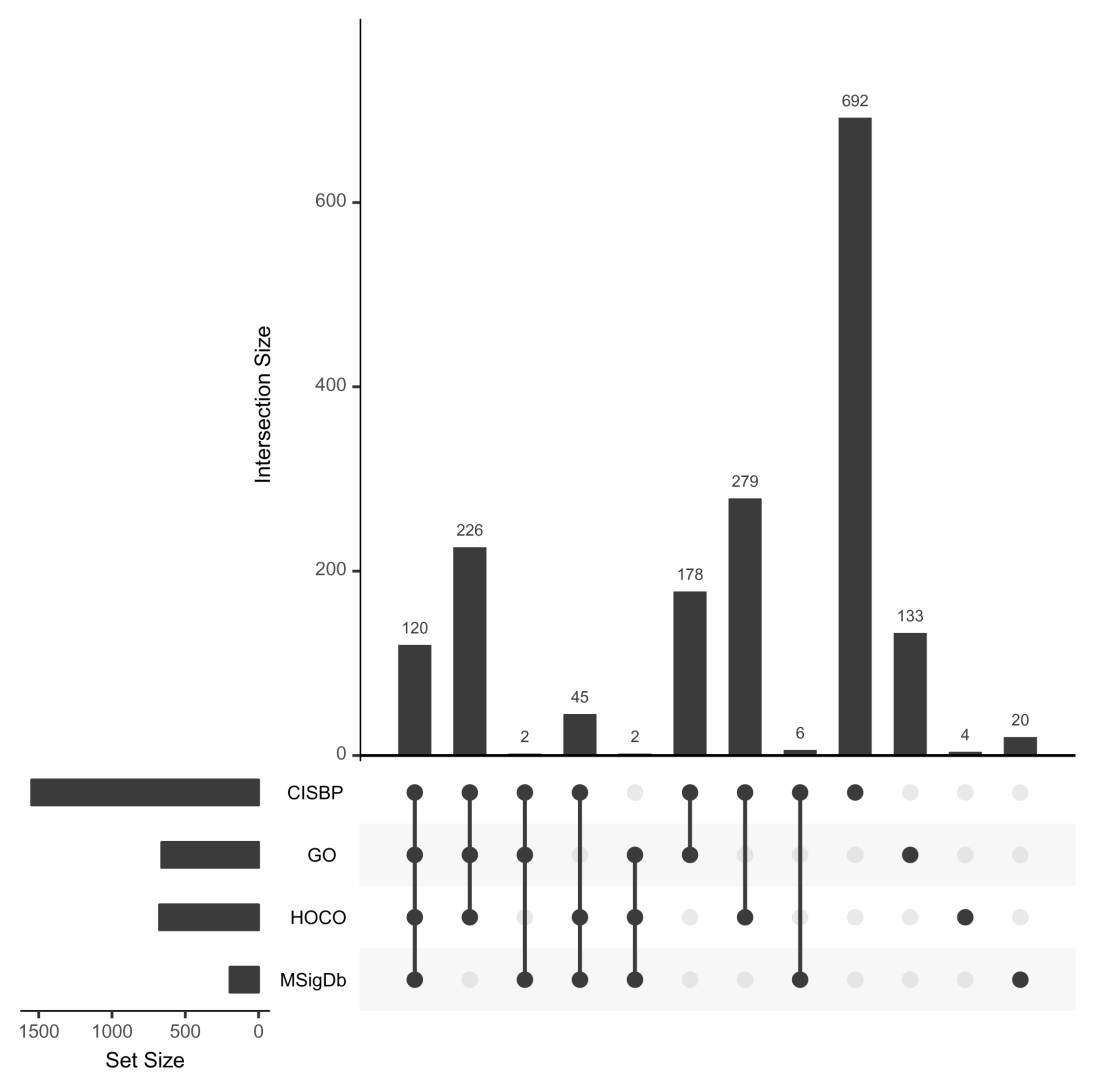
Sizes of transcription factor (TF) catalogs and of intersections based on HGNC (HUGO Gene Nomenclature Committee) symbols for TFs.

In TFutils we have gathered information from four widely used resources, focusing specifically on human TFs: Gene Ontology (GO, Ashburner *et al*. ^3^, in which GO:0003700 is the tag for the molecular function concept “DNA binding transcription factor activity”), CISBP (Catalog of Inferred Sequence Binding Preferences) (Weirauch *et al*. ^2^), HOCOMOCO (Homo sapiens Comprehensive Model Collection) (Kulakovskiy *et al*. ^4^), and the “c3 TFT (transcription factor target)” signature set of MSigDb (Molecular Signatures Database) (Subramanian *et al*. ^5^). Figure 1 depicts the sizes of these catalogs, measured using counts of unique HGNC gene symbols. The enumeration for GO uses Bioconductor’s *org.Hs.eg.db* (version 3.7.0) package to find direct associations from GO:0003700 to HGNC symbols. The enumeration for MSigDb is heuristic and involves parsing the gene set identifiers used in MSigDb for exact or close matches to HGNC symbols. For CISBP and HOCOMOCO, the associated web servers provide easily parsed tabular catalogs.

### Classification of transcription factors

**Figure 2.**
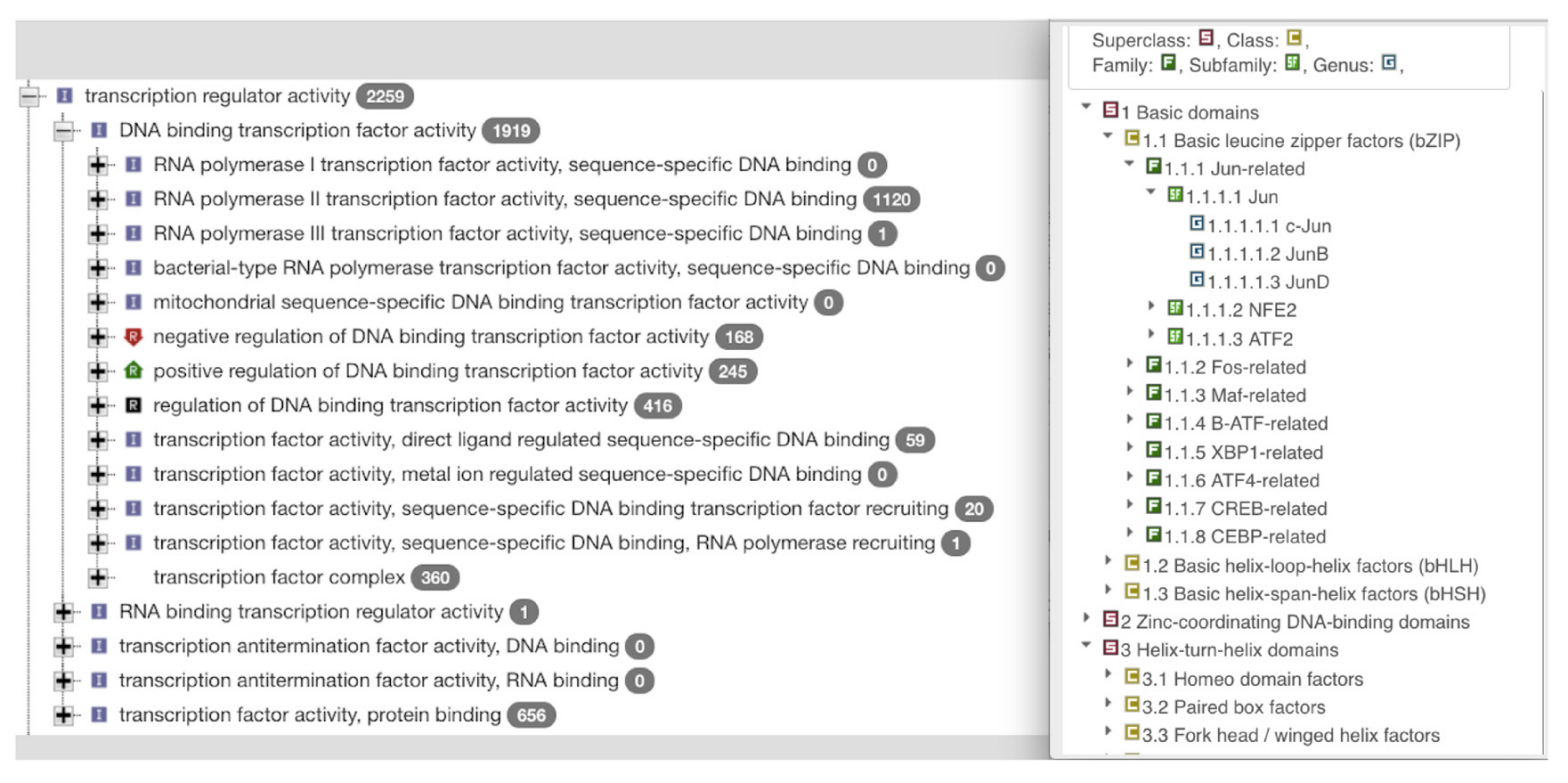
Screenshots of AmiGO and TFClass hierarchy excerpts.

As noted by Weirauch *et al*. ^2^, interpretation of the “function and evolution of DNA sequences” is dependent on the analysis of sequence-specific DNA binding domains. These domains are dynamic and cell-type specific (Gertz *et al*. ^6^). Classifying TFs according to features of the binding domain is an ongoing process of increasing intricacy. Figure 2 shows excerpts of hierarchies of terms related to TF type derived from GO (on the left) and TFclass (Wingender *et al*. ^7^). There is a disagreement between our enumeration of TFs based on GO in Figure 1 and the 1919 shown in AmiGO, as the latter includes a broader collection of receptor activities.

Table 1 provides examples of frequently encountered TF classifications in the CISBP and HOCOMOCO catalogs. The numerical components of the HOCOMOCO classes correspond to TFClass subfamilies (Wingender *et al*. ^7^).

**Table 1.** Most frequently represented transcription factor (TF) classes in CISBP and HOCOMOCO. The number of unique human TF_Name entries in CISBP is 1734. The number of unique Transcription factor entries in HOCOMOCO (Sept. 2018 version) is 678. Entries in columns Nc (Nh) are numbers of distinct TFs annotated to classes in columns CISBP (HO-COMOCO) respectively. Entries are ordered top to bottom by frequency of occurrence. There is no substantive correspondence between entries on a given row. Harmonization of class terminology is beyond the scope of this paper.

CISBP	Nc	HOCOMOCO	Nh
C2H2 ZF	655	More than 3 adjacent zinc finger factors{2.3.3}	106
Homeodomain	199	HOX-related factors{3.1.1}	41
bHLH	104	NK-related factors{3.1.2}	36
bZIP	66	Paired-related HD factors{3.1.3}	35
Unknown	49	Factors with multiple dispersed zinc fingers{2.3.4}	30
Forkhead	48	Forkhead box (FOX) factors{3.3.1}	27
Sox	48	Ets-related factors{3.5.2}	25
Nuclear receptor	46	Three-zinc finger Krueppel-related factors{2.3.1}	20
Myb/SANT	30	POU domain factors{3.1.10}	18
Ets	27	Tal-related factors{1.2.3}	18

### Enumerating TF targets

The Broad Institute MSigDb (Subramanian *et al*. ^5^) includes a gene set collection devoted to cataloging TF targets. We have used Bioconductor’s *GSEABase* package (version 1.45.0) to import and serialize the gmt representation of this collection.


TFutils::tftColl

## GeneSetCollection
##   names: AAANWWTGC_UNKNOWN, AAAYRNCTG_UNKNOWN, ..., GCCATNTTG_YY1_Q6 (615 total)
##   unique identifiers: 4208, 481, ..., 56903 (12774 total)
##   types in collection:
##     geneIdType: EntrezIdentifier (1 total)
##     collectionType: NullCollection (1 total)


Names of TFs for which target sets are assembled are encoded in a systematic way, with underscores separating substrings describing motifs, genes, and versions. Some peculiarity in nomenclature in the MSigDb labels can be observed:


grep("NFK",names(TFutils::tftColl),value=TRUE)

## [1] "NFKAPPAB65_01"         "NFKAPPAB_01"          "NFKB_Q6"
## [4] "NFKB_C"                "NFKB_Q6_01"           "GGGNNTTTCC_NFKB_Q6_01"


Manual curation will be needed to improve the precision with which MSigDb TF target sets can be associated with specific TFs or motifs.

### Quantitative predictions of TF binding affinities

In this subsection we address representation of putative binding sites. First we illustrate how to represent sequence-based affinity measures and the binding site locations implied by these. We then discuss use of results of ChIP-seq experiments for cell-type-specific binding site enumeration.

**Affinity scores based on reference sequence.** The FIMO algorithm of the MEME suite (Grant *et al*. ^8^) was used to score the human reference genome for TF binding affinity for 689 motif matrices to which genes are associated. Full details of the execution of FIMO are provided in Sonawane *et al* ^9^. Sixteen (16) tabix-indexed BED files are lodged in an AWS S3 bucket for illustration purposes.


library(GenomicFiles)
data(fimo16)
fimo16

## GenomicFiles object with 0 ranges and 16 files:
## files: M0635_1.02sort.bed.gz, M3433_1.02sort.bed.gz, ..., M6159_1.02sort.bed.gz, M6497_1.02sort.bed.
## detail: use files(), rowRanges(), colData(), ...



head(colData(fimo16))

## DataFrame with 6 rows and 2 columns
##          Mtag        HGNC
##   <character> <character>
## 1     M0635_1      DMRTC2
## 2     M3433_1       HOXA3
## 3     M3467_1        IRF1
## 4     M3675_1      POU2F1
## 5     M3698_1        TP53
## 6     M3966_1       STAT1


We harvest scores in a genomic interval of interest (bound to fimo16 in the rowRanges assignment below) using reduceByFile. This yields a list with one element per file. Each such element holds a list of scanTabix results, one per query range.


library(BiocParallel)
register(SerialParam())# important for macosx?
rowRanges(fimo16) =GRanges("chr17",IRanges(38.077e6,38.084e6))
rr = GenomicFiles::reduceByFile(fimo16,MAP=function(r,f)
scanTabix(f,param=r))


scanTabix produces a list of vectors of text strings, which we parse with data.table::fread. The resulting tables are then reduced to a genomic location and -log10 of the p-value derived from the binding affinity statistic of FIMO in the vicinity of that location.



asdf =function(x) data.table::fread(paste0(x,collapse="\n"),header=FALSE)
gg =lapply(rr,function(x) {
tmp =asdf(x[[1]][[1]])
data.frame(loc=tmp$V2,score=-log10(tmp$V7))
})
for(iin1:length(gg))  gg[[i]]$tf =colData(fimo16)[i,2]


It turns out there are too many distinct TFs to display names individually, so we label the scores with the names of the associated TF families as defined in CISBP.



matchcis =match(colData(fimo16)[,2], cisbpTFcat[,2])
famn = cisbpTFcat[matchcis,]$Family_Name
for(iin1:length(gg))  gg[[i]]$tffam = famn[i]
nn =do.call(rbind, gg)


**Figure 3.**
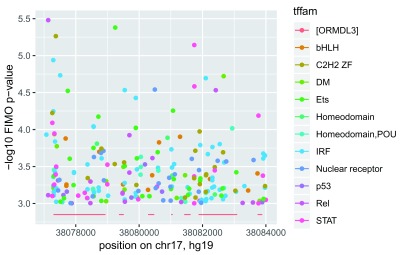
TF binding in the vicinity of gene *ORMDL3*. Points are -log10-transformed FIMO-based p-values colored according to TF class as annotated in CISBP. Segments at bottom of plot are transcribed regions of *ORMDL3* according to UCSC gene models in build hg19.

A simple display of *predicted* TF binding affinity near the gene *ORMDL3* is provided in Figure 3.

**Figure 4.**
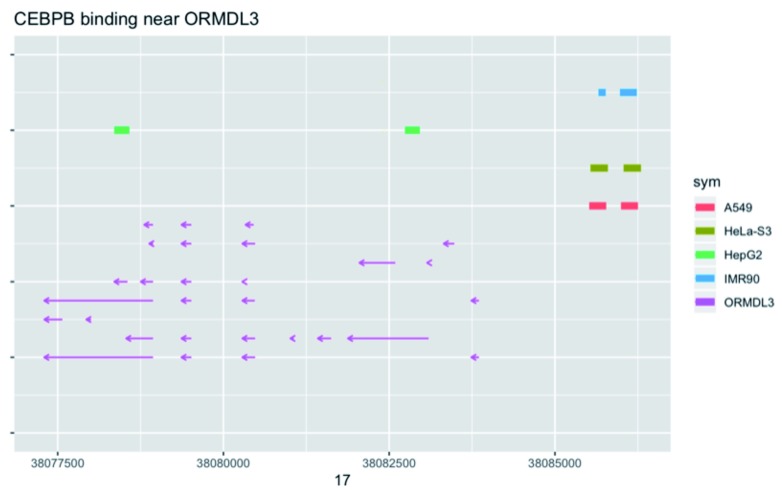
Binding of CEBPB in the vicinity of *ORMDL3* derived from ChIP-seq experiments in four cell lines reported by ENCODE. Colored rectangles at top are regions identified as narrow binding peaks, arrows in bottom half are exons in *ORMDL3*. Arrows sharing a common vertical position are members of the same transcript as cataloged in Ensembl version 75.

**TF binding predictions based on ChIP-seq data from ENCODE.** The ENCODE project provides BED-formatted reports on ChIP-seq experiments for many combinations of cell type and DNA-binding factors. TFutils includes a table encode690 that gives information on 690 experiments involving pairs formed from 91 cell lines and 161 TFs for which results have been recorded as GRanges instances that can be acquired with the *AnnotationHub* (version 2.15.4) package. Positional relationships between cell-type specific binding sites and genomic features can be investigated. An illustration is given in Figure 4, in which is it suggested that in HepG2 cells, CEBPB exhibits a distinctive pattern of binding in the vicinity of *ORMDL3*.

### Visualization of motif relationships in a family of transcription factors 

**Figure 5.**
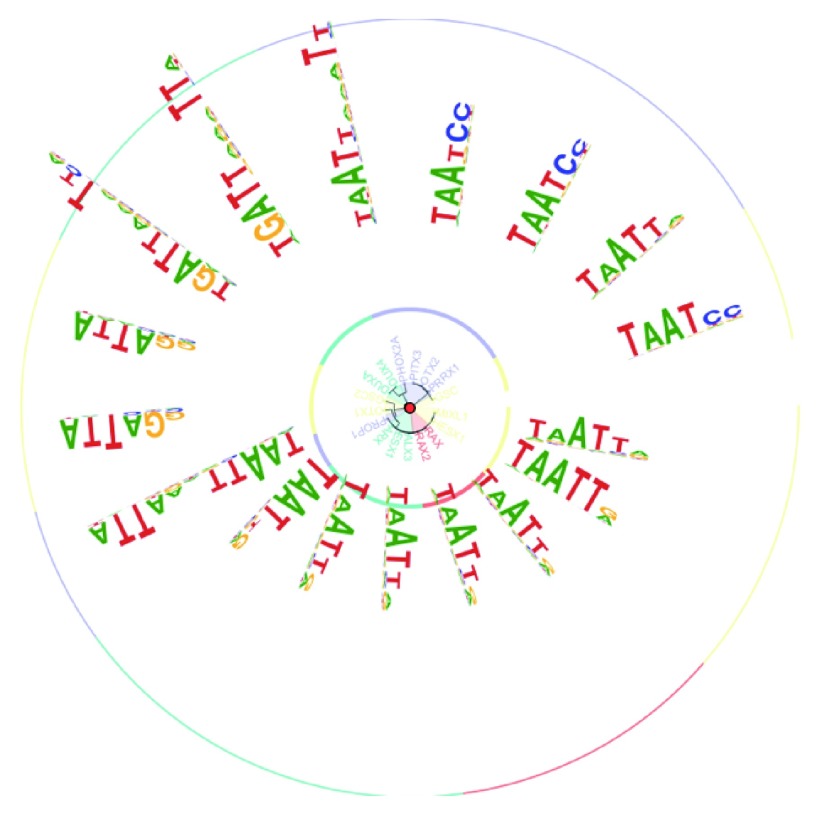
A circos display of motifs of transcription factors in the TFclass 3.1.3 (paired-related homeodomain factors).

Inspired by a referee’s suggestion, we created functions that couple the HOCOMOCO TFclass enumeration with Bioconductor’s MotifDb ^10^ and motifStack ^11^ package resources. Figure 5 is the output of example(tffamCirc.plot), available in version 1.5.1 of TFutils.

### Summary

We have compared enumerations of human transcription factors by different projects, provided access to two forms of binding domain classification, and illustrated the use of cloud-resident genome-wide binding predictions. In the next section we review selected details of data structures and methods of the *TFutils* package.

## Methods

### Implementation

The TFutils package is designed to lower barriers to usage of key findings of TF biology in human genome research. TFutils is supplied as a conventional R package distributed with, and making use of, the Bioconductor software ecosystem. TFutils includes ready-to-use reference data, tools for visualizing binding sites, and tools that simplify integrative use of TF binding information with GWAS findings. A complete enumeration of functions and data available in the package is provided in the reference manual at http://bioconductor.org/packages/release/bioc/manuals/TFutils/man/TFutils.pdf

### Data resources

**Catalogs.** Two reference resources have been collected into the TFutils package as data.frame instances. These are cisbpTFcat (CISBP: 7592 x 28), and hocomoco.mono.sep2018 (mononucleotide models, full catalog, 769 x 9). These data.frames are snapshots of the CISBP and HOCOMOCO catalogs.

**Indexed BED in AWS S3.** As described above fimo16 provides programmatic access to FIMO scores for 16 TFs, using the *GenomicFiles* (version 1.19.0) protocol.

**Annotated reference to ENCODE ChIP-seq results.** encode690 simplifies programmatic access to TF:cell-line combinations available in Bioconductor *AnnotationHub* (version 2.15.4).

**TF targets enumerated in MsigDb.** The c3-TFT (TF targets) subset from MSigDb is provided as a GeneSet-Collection instance as defined in *GSEABase*.

**Illustrative GWAS records.** The full EBI/EMBL GWAS catalog is available in the *gwascat* package (version 2.15.0); for convenience, an excerpt focusing on chromosome 17 is supplied with TFutils as gwascat_hg19_chr17.

### Infrastructure for interacting with components of TFutils

**Figure 6.**
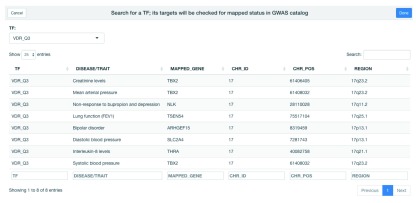
TFtargs screenshot. This example reports on recent EBI GWAS catalog hits on chromosome 17 only.

**Interactive enumeration of TF targets implicated in GWAS.** The TFtargs function runs a shiny app that permits selection of a TF in the nomenclature of the MSigDb c3/TFT gene set collection. The app will search an object provided by the *gwascat* package for references in the MAPPED_GENE field that match the targets of the selected TF. Figure 6 gives an illustration.

**The TFCatalog S4 class**. Reference catalogs for TF biology are structured with the TFCatalog S4 class. Two essential components for managing a catalog are the native TF identifier for the catalog and the HGNC gene symbol typically used to name the TF. The TFCatalog class includes a name field to name the catalog, and a character vector with elements comprised of the native identifiers for catalogued TFs.

For example, CISBP uses T004843_1.02 to refer to motifs associated with gene TFAP2B. There are five such motifs, three derived from SELEX, one from Transfac, and one from Hocomoco.

A data.frame instance that has an obligatory column named ‘HGNC’ can include any collection of fields that offer metadata about the TF in the specified catalog. Here is how we construct and view a TFCatalog object using the CISBP reference data.


data(cisbpTFcat)
TFs_CISBP =TFCatalog(name="CISBP.info",
nativeIds=cisbpTFcat[,1],
HGNCmap =cisbpTFcat)
TFs_CISBP

## TFutils TFCatalog instance CISBP.info
##  7592 native Ids, including
##    T004843_1.02 ... T153733_1.02
##  1551 unique HGNC tags, including
##    TFAP2B TFAP2B ... ZNF10 ZNF350


### Operation: Installation

The TFutils package can be installed in any version of R subsequent to 3.5.0, and therefore will be usable on Unix, Windows, or Mac platforms. The preferred method of installation employs the CRAN package BiocManager, through the R command BiocManager::install("TFutils"). All necessary dependencies will be installed through this process.

### Operation: Use cases

In this section we consider applications of the tools in genetic epidemiology. First we look for TFs that may harbor variants associated with traits in the EBI GWAS catalog. Then we show how to enumerate traits associated with targets of a selected TF.

**Find TFs that are direct GWAS hits for a given trait.** directHitsInCISBP accepts a string naming a trait, and returns a data.frame of TFs identified as “mapped genes” for the trait, with their TF “family name”.


library(dplyr)
library(magrittr)
library(gwascat)
data(ebicat37)
directHitsInCISBP(
                        "Rheumatoid arthritis", ebicat37)

## Joining, by = "HGNC"

##      HGNC Family_Name
## 1  ARID5B ARID/BRIGHT
## 7   EOMES       T-box
## 15  GATA3        GATA
## 35  JAZF1     C2H2 ZF
## 37  MECP2         MBD
## 45   MTF1     C2H2 ZF
## 57    REL         Rel
## 65  STAT4        STAT
## 79   AIRE        SAND
## 82   IRF5         IRF


**Retrieve traits mapped to genes that are targets of a given TF.** topTraitsOfTargets will acquire the targets of a selected TF, check for hits in these genes in a given GWAS catalog instance, and tabulate the most commonly reported traits.


tt =topTraitsOfTargets("MTF1", TFutils::tftColl, ebicat37)

## remapping identifiers of input GeneSetCollection to Symbol...

## done

head(tt)

##                              DISEASE.TRAIT MAPPED_GENE       SNPS CHR_ID
## 1                        Atopic dermatitis        TNXB rs41268896      6
## 2                        Atopic dermatitis        TNXB rs12153855      6
## 3                        Atopic dermatitis       KIF3A  rs2897442      5
## 4 Attention deficit hyperactivity disorder      SEMA3A   rs797820      7
## 5 Attention deficit hyperactivity disorder        DNM1  rs2502731      9
## 6 Attention deficit hyperactivity disorder        GPC6  rs7995215     13
##     CHR_POS
## 1  32102292
## 2  32107027
## 3 132713335
## 4  83979723
## 5 128214278
## 6  93756253

table(tt[,1])

##
##                        Atopic dermatitis
##                                        3
## Attention deficit hyperactivity disorder
##                                        3
##                                   Height
##                                        7
##                  Menarche (age at onset)
##                                        4
##                   Obesity-related traits
##                                       11
##                     Rheumatoid arthritis
##                                        3


## Discussion

Sources and consequences of variations in DNA transcription are fundamental problems for cell biology, and the projects we have made use of for cataloging transcription factors are at the boundaries of current knowledge.

It is noteworthy that the four resources used for Figure 1 agree on names of only 119 TFs. The fact that CISBP distinguishes 475 TFs that are not identified in any other source should be better understood. We observe that the ascription of TF status to AHRR is based on its sharing motifs with AHR (see http://cisbp.ccbr. utoronto.ca/TFreport.php?searchTF=T014165_1.02).

Figure 2 and Table 1 show that the classification of TFs is now fairly elaborate. Use of the precise terminology of the TFClass system to label TFs of interest at present relies on associations provided with the HOCOMOCO catalog.

As population studies in genomic and genetic epidemiology grow in size and scope, principles for organizing and prioritizing loci associated with phenotypes of interest are urgently needed. Figure 6 shows that loci associated with phenotypes related to kidney function, lung function, and IL-8 levels are potentially unified through the fact that the GWAS hits are connected with genes identified as targets of VDR (vitamin D receptor). This example limited attention to hits on chromosome 17; the TFtargs tool permits *ad libitum* exploration of phenotype-locus-gene-TF associations. Our hope is that the tools and resources collected in TFutils will foster systematic development of evidence-based mechanistic network models for transcription regulation in human disease contexts, thereby contributing to the development of personalized genomic medicine.

## Data availability

With the exception of the FIMO scoring data ( fimo16), all data underlying the results are available as part of the article and no additional source data are required.

fimo16 links to indexed bed files in a public S3 bucket funded by the Bioconductor foundation. The underling data is sourced from Sonawane *et al*. 2017 https://doi.org/10.1016/j.celrep.2017.10.001 ^9^

## Software availability

Source code is available from GitHub: https://github.com/vjcitn/TFutils

Archived source code: https://doi.org/doi:10.18129/B9.bioc.TFutils ^12^

Licence: Artistic License 2.0
