# Supplementary material for: TFutils: Data structures for transcription factor bioinformatics
Source: F1000Res. 2019 May 17;8:152. Originally published 2019 Feb 5. [Version 2] doi: 10.12688/f1000research.17976.2 (PMC6600865; doi:10.12688/f1000research.17976.2)
Supplement: Peer review history

# Peer review: Reviewer response for version 2

**DOI:** 10.5256/f1000research.21137.r49980

**Published:** 2019-07-18

**Authors:** Matthew T. Weirauch, Kevin Ernst

**Affiliations:** 1 Cincinnati Children's Hospital Medical Center, Cincinnati, OH, USA; 2 Center for Autoimmune Genomics and Etiology, Cincinnati Children's Hospital Medical Center (CCHMC) , Cincinnati, OH, USA

The authors present TFutils, a Bioconductor package for analyze TFs and their binding sites. TFutils combines information and analysis capabilities from several popular sources into one interface. The authors present use-cases and tutorials for how TFutils can be used to ask and answer several biological questions.

 Major Comments

1. From what I can tell, the manuscript is basically a copy-pasted version of the “vignette” from their TFutils R package on Bioconductor, probably written by the (co)author of the package.  If the purpose is to teach people how to use their TFutils package, or even compare/contrast it with other existing solutions, in my opinion the manuscript falls very short of this goal. It’d be different if the vignette (or the paper) made a clear case for what TFutils does right up front. Apart from saying it doesn’t do sequence logo visualizations, the introduction lacks any discussion of what good the package is, except that it “assembles various resources intended to clarify and unify approaches to working with TF concepts in bioinformatic analysis”. A helpful remedy for this would be to have a table of included functions, data structures, and sample datasets, similar to the auto-generated listing from their own package, which is already publicly-available on the web.  The “Intro” section (actually the whole paper) lacks any straightforward enumeration of the contents of the package like “we include data structures (name them) that wrap X, Y, and Z databases, and functions alpha, beta, and gamma to do (whatever).”  
2. The manuscript itself is very hard to follow. It would be much better if there were with a common, practical theme through the examples – as written, they are quite disjoint and not very well explained. There is no unifying thread to any of these examples, no focus on telling a “story” with a real-life research inquiry, or making the case for how TFutils could make that inquiry easier. If people in my lab (a transcription factor bioinformatics lab) have trouble following it, then there is almost no chance that a non-expert could follow it. One thing that would really help would be more human language in the code samples parts (or code comments), in order to aid in reader understanding. As it stands, the text surrounding the code is choppy, and does little to illuminate what’s going on in the code samples. The examples and the text surrounding them are sometimes painfully disjoint, to the point where I was wondering how the text and code and figures right next to each other were related.
3. I would strongly urge the authors to use the Lambert *et al. *collection of 1,639 human TFs as their basis. Bigger is not always better. Although the other databases currently used collectively sum to many more human TFs, there are many false positives. For example, GO includes things like kinases in this category. This is exactly the reason that Lambert and colleagues went to the painstaking efforts of collecting all human TF candidates and manually curating the list one by one.
4. Table 1 is comparing apples to oranges. Cis-BP contains all human TFs, regardless of their motif status (i.e. even if a TF does not have a known DNA binding motif, it is still included in the database). HOCOMOCO, on the other hand, only includes TFs with motifs. So, it does not really make sense to compare their TF members, which seems to be the major point of Table 1. This also applies to the following comment in the Discussion, which also does not really make sense based on these facts: “is noteworthy that the four resources used for Figure 1 agree on names of only 119 TFs. The fact that CIS-BP distinguishes 475 TFs that are not identified in any other source should be better understood. We observe that the ascription of TF status to AHRR is based on its sharing motifs with AHR.” The only reason AHRR is not in the other databases is probably because it has not had its motif directly determined through experimentation.
5. The example functionality shown in Figure 6 does not make sense to me. As I understand it, a “TF target” is from MSigDB, which is simply a predicted binding site for a TF (here, VDR), in the promoter of a gene (e.g., TBX2 in the top row of the figure). TBX2 is associated with, e.g. Creatine Levels. But, we do not know where the GWAS signal is located relative to TBX2. The GWAS signal could be 20,000 bases away from TBX2, or inside its intron, etc. So, what is the connection here between binding of VDR to the promoter of TBX2 and the GWAS signal? Unless I am misunderstanding how this part of the tool is working, this analysis seems very misleading to me.

Minor comments

1. Introduction: “typically near gene promoter regions” – I would add “and enhancers”, since this is actually where the majority of the TF binding sites are located.
2. There was just a new release of Cis-BP (version 2.0) – if its not too much work, I would urge the authors to update their system, since it is a major update. I realize that this can be a lot of work, so I will leave it to the authors to decide if this is worth immediate action.
3. I would be very careful about calling the MSigDB collection “TF targets” – these are not experimentally determined binding events (e.g., through ChIP-seq). These are simply the result of scanning motifs in promoters. I think these should therefore only be referred to as “predicted targets”. This seems nitpicky, but I think there is a very important distinction here.
4. It looks like the motifs from FIMO are Cis-BP motifs (these are incorporated into FIMO), which is fine. But according to the example shown, it looks like the IDs might be truncated in your database – for example, the top one is called “M3433_1”, which could lead to ambiguities, since it could correspond to “M3433_1.02”, “M3433_1.01”, “M3433_1.00”, etc.
5. It is usually not mentioned whether a function or data structure comes from TFutils or some other R package - a simple inline comment or note in the accompany text would really clear this up.
6. Dependencies are left up to the reader to figure out, which is a bit of a nuisance for someone that is trying to decide whether or not a given package is worth exploring.
7. Cis-BP should be spelled “Cis-BP” (as it is in Determination and inference of eukaryotic transcription factor sequence specificity - Weirauch *et al. *(2014) ^1^).
8. Some of the sentence structure and word choices (data is “lodged” in Amazon S3, “harvesting” insights from the data) are inappropriate.
9. There are numerous “typesetting” problems in the paper, most of which, I assume originate with the original R Markdown vignette.
10. Monospace font not used consistently for variable or function names (numerous occurrences) - in many places throughout the text.
11. A bold font is used where a 4th-level headline (####) should be, which when rendered as HTML, creates a run-on with the first sentence of the intended section; e.g.:
  Annotated reference to ENCODE ChIP-seq results.encode690
  Find TFs that are direct GWAS hits for a given trait.directHitsInCISBP
12. Additional individual nit-picks (there are many) can be found in a marked-up version of v2 of the paper, found here. 

We confirm that we have read this submission and believe that we have an appropriate level of expertise to state that we do not consider it to be of an acceptable scientific standard, for reasons outlined above.

## References

[1] : Determination and inference of eukaryotic transcription factor sequence specificity. *Cell*.2014;158(6) : 10.1016/j.cell.2014.08.009 1431-1443 10.1016/j.cell.2014.08.009 25215497PMC4163041

# Peer review: Reviewer response for version 2

**DOI:** 10.5256/f1000research.21137.r48671

**Published:** 2019-06-27

**Authors:** Philipp Bucher, Giovanna Ambrosini

**Affiliations:** 1 Swiss Institute for Experimental Cancer Research (ISREC), School of Life Sciences, Swiss Federal Institute of Technology (EPFL), Lausanne, Switzerland

The authors have followed up on several of our suggestions and this (in our opinion) has significantly improved the article.

We confirm that we have read this submission and believe that we have an appropriate level of expertise to confirm that it is of an acceptable scientific standard.

# Peer review: Reviewer response for version 2

**DOI:** 10.5256/f1000research.21137.r48672

**Published:** 2019-05-28

**Authors:** Lihua Julie Zhu, Rui Li

**Affiliations:** 1 Department of Molecular, Cell and Cancer Biology, University of Massachusetts Medical School (UMMS), Worcester, MA, USA; 2 University of Massachusetts Medical School, Worcester, MA, USA

We think that the authors have addressed our major concerns, and we would like to change the status to Approved for the revised version.

 There are a couple of typos in the revised version that the authors might want to correct.

1. Figure 4 legend states that there are four cell lines while the figure seems to depict data for 5 cell lines.
2. The authors probably meant “four” instead of “for” in the following sentence under the Discussion section.
  Sources and consequences of variations in DNA transcription are fundamental problems for cell biology, and the projects we have made use of **for** cataloging transcription factors are at the boundaries of current knowledge.”

We confirm that we have read this submission and believe that we have an appropriate level of expertise to confirm that it is of an acceptable scientific standard.

# Peer review: Reviewer response for version 1

**DOI:** 10.5256/f1000research.19660.r45003

**Published:** 2019-04-26

**Authors:** Philipp Bucher, Giovanna Ambrosini

**Affiliations:** 1 Swiss Institute for Experimental Cancer Research (ISREC), School of Life Sciences, Swiss Federal Institute of Technology (EPFL), Lausanne, Switzerland

TFutils is a Bioconductor package meant to help users study TF binding in the human genome. The tool integrates several resources such as Gene Ontology (GO), CISBP, HOCOMOCO, and MSigD (the Molecular Signature Database). The paper describes how to tackle basic problems users are faced with when trying to work with TFs, in particular the TF classification, the gene targets identification, and, ultimately, the prediction of TF binding affinities.

 This article looks more like a software tutorial than a scientific article. As software has to evolve in order keep up with user needs, the text will have to be updated on a regular basis in the future, in order to remain up-to-data as well. This is fine if F1000Research accepts updates and supports versioning of articles. Otherwise, another format should be chosen for presenting this tool.

 Just by reading the article, we didn't get a clear impression of what is inside TFutils. Going through the command examples was helpful in this respect. Nevertheless, we have doubts whether we would be able to use this package in a productive manner in the future. The promise that "TFutils lowers the barriers of usage of key findings of TF biology" holds only for expert users of Bioconductor, who are already familiar with all the other packages mentioned in this article and necessary to reproduce the results.

 The current manuscript has several shortcomings. At a general level, it is not very transparent to the naïve reader what is actually new from this package and what functionalities are provided by the many other Bioconductor packages referred to in the text. Fortunately, we found a well-organized reference manual for TFutils version 1.2.0 on the internet, which clarified this issue for us. A URL to this document should have been included in the article.

 As this is a tutorial-style document, it would be helpful to provide complete R code for reproducing Figures 3 and 4. While Figure 3 is relatively easy to generate, it took us at least half a day to reproduce Figure 4. A major limitation is that the fimo16 object, upon which Figure 3 is based, contains only TF affinity data for 16 out of 689 scanned TF motif matrices.

 Figure 3 shows the predicted binding sites for 16 TFs in a selected genomic region. Already with such a small number of TFs, the Figure is pretty crowded with dots. One wonders what it would look like if all 689 FIMO-scanned motif matrices were considered. In view of the density of motif matches it seems doubtful whether any biological insights can be gained from such a plot. Some guidance for the interpretation is needed.

 Figure 4 shows ENCODE binding peaks for CEBPB in the same genomic region that was used for Figure 3. Naturally, we were curious to know whether the peaks seen in this Figures co-localize with corresponding motif matches in Figure 3. Unfortunately, CEBPB is not included in the fimo16 collection. To exemplify the power of the tool, it would have been preferable to choose an example where the reader can crosscheck the consistency between predictions and experiments via comparison of Figure 3 with Figure 4.

 Overall, our impression is that TFutils is a useful package albeit for a restricted community of users already familiar with the other Bioconductor packages mentioned in the article. However, the manuscript could benefit from major revisions as pointed out above.

We confirm that we have read this submission and believe that we have an appropriate level of expertise to confirm that it is of an acceptable scientific standard, however we have significant reservations, as outlined above.

# Response

**Published:** 2019-05-08

**Authors:** Vincent Carey

**Affiliations:** Harvard University, USA

We are very appreciative of the effort underlying this review.  Important questions were raised and we endeavor to answer them fully below.  Reviewer comments are prefaced by " **QUERY**" and our replies are prefaced by " **RESPONSE**".

**QUERY**: This article looks more like a software tutorial than a scientific article. As software has to evolve in order keep up with user needs, the text will have to be updated on a regular basis in the future, in order to remain up-to-data as well. This is fine if F1000Research accepts updates and supports versioning of articles. Otherwise, another format should be chosen for presenting this tool.

**RESPONSE**:  Because F1000Research accepts updates and supports versioning of articles, we believe that this format is an acceptable one for the work we have described.

**QUERY**: Just by reading the article, we didn't get a clear impression of what is inside TFutils. Going through the command examples was helpful in this respect. Nevertheless, we have doubts whether we would be able to use this package in a productive manner in the future. The promise that "TFutils lowers the barriers of usage of key findings of TF biology" holds only for expert users of Bioconductor, who are already familiar with all the other packages mentioned in this article and necessary to reproduce the results.

**RESPONSE**: We are glad that the examples in the paper were useful to the reviewer. It is true that Bioconductor software generally requires acquaintance with and use of multiple interrelated packages. In this sense, it is different from a relatively common approach of command-line utility implementation of bioinformatic analysis tools, where a given tool may be understood and used in isolation. We do not agree that "expert" level of understanding of Bioconductor is necessary to make use of the material described, although some facility with and enthusiasm for the R language would be necessary to make headway. The paper is published in the Bioconductor channel of F1000research, and it is expected that the readership will have an acquaintance with the resources and limitations of the Bioconductor software and data ecosystem.

**QUERY**: The current manuscript has several shortcomings. At a general level, it is not very transparent to the naïve reader what is actually new from this package and what functionalities are provided by the many other Bioconductor packages referred to in the text. Fortunately, we found a well-organized reference manual for TFutils version 1.2.0 on the internet, which clarified this issue for us. A URL to this document should have been included in the article.

**RESPONSE**: This is a useful observation. We have now included a reference to http://bioconductor.org/packages/release/bioc/manuals/TFutils/man/TFutils.pdf

in the implementation section of the paper.

**QUERY**: As this is a tutorial-style document, it would be helpful to provide complete R code for reproducing Figures 3 and 4. While Figure 3 is relatively easy to generate, it took us at least half a day to reproduce Figure 4. A major limitation is that the fimo16 object, upon which Figure 3 is based, contains only TF affinity data for 16 out of 689 scanned TF motif matrices.

**RESPONSE**: We appreciate the effort taken here. The production of Figure 4 was complicated and we have created an app and associated github repository to clarify the basic issues. The repository is https://github.com/vjcitn/encdemo and the face page of the repo has a screenshot of the app, which runs at https://vjcitn.shinyapps.io/encdemo/. Our point in the visualization of Figure 4 is not the visualization per se, which can be accomplished with standard genome browsers, with suitable commands. Rather, we use the visualization to give concrete demonstration of the immediate programmatic availability (to users of this package) of the relevant experimental results and annotations. We concur that the limitation of fimo16 to a small number of TFs is disappointing. Comprehensive presentation of the scan scores to our user base/readership would require computational resources that we have not yet been able to muster. A local deployment requires close to a terabyte of indexed storage.

**QUERY**: Figure 3 shows the predicted binding sites for 16 TFs in a selected genomic region. Already with such a small number of TFs, the Figure is pretty crowded with dots. One wonders what it would look like if all 689 FIMO-scanned motif matrices were considered. In view of the density of motif matches it seems doubtful whether any biological insights can be gained from such a plot. Some guidance for the interpretation is needed.

**RESPONSE**: As noted just above we do not have a mechanism for providing all 689 scans. We have added text after Figure 3 acknowledging the challenge of interpretation, specifically with respect to combinatorics of TF binding.

**QUERY**: Figure 4 shows ENCODE binding peaks for CEBPB in the same genomic region that was used for Figure 3. Naturally, we were curious to know whether the peaks seen in this Figures co-localize with corresponding motif matches in Figure 3. Unfortunately, CEBPB is not included in the fimo16 collection. To exemplify the power of the tool, it would have been preferable to choose an example where the reader can crosscheck the consistency between predictions and experiments via comparison of Figure 3 with Figure 4.

**RESPONSE**: We agree that unification of concepts underlying Figure 3 and 4 would be quite desirable. Figure 3 is based on the analysis of motifs in reference sequence, while Figure 4 is a severe reduction of cell-type specific information from in vitro experiments. The data underlying the encdemo app noted above should be useful for beginning surveys across TFs and across cell types essential for a full understanding of cell-type specific combinatorics of TF binding.

**QUERY**: Overall, our impression is that TFutils is a useful package albeit for a restricted community of users already familiar with the other Bioconductor packages mentioned in the article. However, the manuscript could benefit from major revisions as pointed out above.

**RESPONSE**: We appreciate the effort taken in this review and we have endeavored to answer the questions raised.

# Peer review: Reviewer response for version 1

**DOI:** 10.5256/f1000research.19660.r44033

**Published:** 2019-02-20

**Authors:** Lihua Julie Zhu, Haibo Liu, Rui Li

**Affiliations:** 1 Department of Molecular, Cell and Cancer Biology, University of Massachusetts Medical School (UMMS), Worcester, MA, USA; 2 Iowa State University, Ames, IA, USA; 3 University of Massachusetts Medical School, Worcester, MA, USA

The TFutils package provides useful, convenient, integrated data structures for TF-related bioinformatics analyses, by incorporating the basic information of human transcription factors (TFs), such as TF classification, known TF targets, genome-wide TF binding sites and binding affinity scores, which might be used to prioritize candidate genetic variants and help understand gene transcriptional regulatory mechanisms. Importantly, it also provides an interactive interface to query TFs and TF targets implicated in human traits as discovered by many GWASs.

 In a quick test, all demo code in this paper worked. However, to make sure TFutils is more useful to the bioinformatics community, a few questions may need to be addressed. Here are our detailed comments and questions.

  

1. TFutils includes resources from CISBP, HOCOMOCO, GO and MSigDb. There are additional human TF resources. Is there any reason not to include those resources such as JASPAR ^1^, Transfac ^2^, HDPI ^3^ and uniPro ^4^? 
2. There are potential packages that will likely import TFutils such as TFBSTools for the analysis of transcription factor binding sites manipulation, motifStack for graphic representation of multiple motifs ^5^ and MotIV. It will be helpful to present a few lines of code to show how to integrate data from TFutils to aforementioned pipelines.
3. The section “Basic concepts of transcription factor bioinformatics” includes lots of background information, such as existing TF-related data sources/bases, TF classification, and how TFutils incorporates and access those resources. To make it easy to follow, we suggest break this part into the Introduction section and the Method section. The author may move the background information and TF classification to the Introduction section, and include an Implementation section in the Methods section to describe how TFutils incorporates all these data sources and how to retrieve the relevant information in TFutils and how to integrate with other packages as mentioned in 2, where the R script snippets can be displayed.
4. To maintain/increase the user base, it is important to keep the data up to date. Currently, the data were snapshots of the CISBP and HOCOMOCO catalogs. If the resources are not updated regularly, it’s unlikely that users will use TFutils after 2-3 years. Is there a plan in place to have the resources assembled by TFutils be update regularly? How often is the update going to be? Is it going to be automatic or manually?
5. Flexibility of the data structure is also important, as users may want to expand the utility of TFutils. Suggest authors describe how to add features to the current data structures in TFutils in the manuscript.
6. It will be useful to add information on the numbers of TFs and targets included in the assembled resources, as well as in the original databases.
7. There is a python package having the same name “tfutils” which is very popular. If it is not too hard to do, we suggest authors change the package name to avoid confusion
8. Installation and running environments of the TFutils was described twice, once in the Introduction section, the other time in the Methods section: Operation: Installation. It is better to only describe this once in the Methods section.
9. There are many short paragraphs consisting of one or two sentences and related information are scattered into different sections. For instances, the last paragraph of the Introduction section about the limitations of TFutil might be moved to the Discussion part; whereas the third paragraph in the Discussion section might be moved to somewhere at the beginning of the Introduction section or where it is appropriate.
10. Page 6, the Summary section might be better moved to between the data availability section and the discussion section to summarize the implemented functionality of TFutil.

Besides those major issues, we also have a few minor questions:

1. Currently the abstract only mentions TF targets derived from the MSigDb. Considering that the ENCODE TF ChIP-seq data is one of the most significant resources for TF targets information as mentioned in the main text, suggest authors add how the ENCODE ChIP-seq data were incorporated into TFutils in the abstract.
2. Page 5, please clarify the type of details in the sentence “Full details are provided in Sonawane et al”.
3. Gene structure can be better depicted in Fig. 3 and Fig. 4, perhaps adopting the gene structure visualization in most genome viewers, showing exon/intron structure and gene transcription direction.
4. Please include the used R packages in the citation.
5. “TFtargs()” in Figure 5 legend needs to be edited.
6. For the subtitles under the Use cases section, suggest add find before “TFs that are direct GWAS …” and retrieve before “Traits mapped to genes that …”.

We confirm that we have read this submission and believe that we have an appropriate level of expertise to confirm that it is of an acceptable scientific standard, however we have significant reservations, as outlined above.

## References

[1] : JASPAR: an open-access database for eukaryotic transcription factor binding profiles. *Nucleic Acids Res*.2004;32(Database issue) : 10.1093/nar/gkh012 D91-4 10.1093/nar/gkh012 14681366PMC308747

[2] : TRANSFAC: an integrated system for gene expression regulation. *Nucleic Acids Res*.2000;28(1) :316-9 1059225910.1093/nar/28.1.316PMC102445

[3] : hPDI: a database of experimental human protein-DNA interactions. *Bioinformatics*.2010;26(2) : 10.1093/bioinformatics/btp631 287-9 10.1093/bioinformatics/btp631 19900953PMC2804296

[4] : UniPROBE: an online database of protein binding microarray data on protein-DNA interactions. *Nucleic Acids Res*.2009;37(Database issue) : 10.1093/nar/gkn660 D77-82 10.1093/nar/gkn660 18842628PMC2686578

[5] : motifStack for the analysis of transcription factor binding site evolution. *Nat Methods*.2018;15(1) : 10.1038/nmeth.4555 8-9 10.1038/nmeth.4555 29298290

# Response

**Published:** 2019-05-08

**Authors:** Vincent Carey

**Affiliations:** Harvard University, USA

We appreciate the careful reading of this paper, which has inspired additional functionality that will be added to the package. Reviewer comments (isolated with **QUERY**: tag) are addressed below (with **RESPONSE**: tag).

**QUERY**: TFutils includes resources from CISBP, HOCOMOCO, GO and MSigDb. There are additional human

TF resources. Is there any reason not to include those resources such as JASPAR1, Transfac2, HDPI3 and uniPro4?

**RESPONSE**: According to http://cisbp.ccbr.utoronto.ca/summary.php?by=4&orderby=MSource_Identifier JASPAR and TRANSFAC entries are included in CIS-BP. UniProbe is an interesting resource but does not provide a mapping from TF or motif to target genes. Interfacing to UniProbe is beyond the scope of the tasks intended for this paper. The same issue appears to us to apply to hPDI.  Resources that do not enumerate "gene level" TF targets are beyond the scope of current work.

**QUERY**: There are potential packages that will likely import TFutils such as TFBSTools for the analysis of transcription factor binding sites manipulation, motifStack for graphic representation of multiple motifs5 and MotIV. It will be helpful to present a few lines of code to show how to integrate data from TFutils to aforementioned pipelines.

**RESPONSE**: We will add an example of how diversity in TF reference motif data leads

to a structure that may be visualized with MotifDb/motifStack. Specifically the 1.5.x+ versions of TFutils will include a function that uses motifStack and MotifDb to generate displays like Figure 5 of the revised paper. This is the result of example(tffamCirc.plot)in the current devel branch of TFutils.

**QUERY**: The section “Basic concepts of transcription factor bioinformatics” includes lots of background information, such as existing TF-related data sources/bases, TF classification, and how TFutils incorporates and access those resources. To make it easy to follow, we suggest break this part into the Introduction section and the Method section. The author may move the background information and TF classification to the Introduction section, and include an Implementation section in the Methods section to describe how TFutils incorporates all these data sources and how to retrieve the relevant information in TFutils and how to integrate with other packages as mentioned in 2, where the R script snippets can be displayed.

**RESPONSE**: Some of these changes are made. Our approach to the narrative attempts to follow the F1000Research schema.

**QUERY**:  To maintain/increase the user base, it is important to keep the data up to date. Currently, the data were snapshots of the CISBP and HOCOMOCO catalogs. If the resources are not updated regularly, it’s unlikely that users will use TFutils after 2-3 years. Is there a plan in place to have the resources assembled by TFutils be update regularly? How often is the update going to be? Is it going to be automatic or manually?

**RESPONSE**: The package will be updated according to the Bioconductor release

protocol. The main pages give explicit information on provenance of information underlying serialized data structures. Community input on the utility of the various sources will be important in determining the frequency of content updates.

**QUERY**: Flexibility of the data structure is also important, as users may want to expand the utility of TFutils. Suggest authors describe how to add features to the current data structures in TFutils in the manuscript.

**RESPONSE**: The code is open source. Pull requests are welcome. If there are specific features of interest to the reviewers we will consider how to incorporate them in future versions of the package/manuscript.

**QUERY**: It will be useful to add information on the numbers of TFs and targets included in the assembled resources, as well as in the original databases.

**RESPONSE**: Totals are added in the caption of Figure 1.

**QUERY**: There is a python package having the same name “tfutils” which is very popular. If it is not too hard to do, we suggest authors change the package name to avoid confusion

**RESPONSE**: The python package addresses "tensorflow", which is not related to TFs

in bioinformatics. We do not believe that the risk of confusion is high, but will consider renaming if events of confusion are observed.

**QUERY**: Installation and running environments of the TFutils was described twice, once in the Introduction section, the other time in the Methods section: Operation: Installation. It is better to only describe this once in the Methods section.

**RESPONSE**: The presentation follows the suggested F1000Research format.  

**QUERY**: There are many short paragraphs consisting of one or two sentences and related information are scattered into different sections. For instances, the last paragraph of the Introduction section about the limitations of TFutil might be moved to the Discussion part; whereas the third paragraph in the Discussion section might be moved to somewhere at the beginning of the Introduction section or where it is appropriate.

Page 6, the Summary section might be better moved to between the data availability section and the discussion section to summarize the implemented functionality of TFutil.

**RESPONSE**: The last paragraph of the introduction is used to pre-empt potential reader disappointment early in the presentation, so we prefer to leave it where it is. The third paragraph in the discussion does include content of general and possibly introductory significance, but that content is embedded in concrete illustrations that depend upon actual details of package use. The "summary" element is provided to give the reader a break before plunging into the obligatory Methods/Implementation material. We concur with your basic aesthetic preferences but have organized our text in what we consider to be a rational way.

**QUERY**: Besides those major issues, we also have a few minor questions:

**QUERY**: Currently the abstract only mentions TF targets derived from the MSigDb. Considering that the ENCODE TF ChIP-seq data is one of the most significant resources for TF targets information as mentioned in the main text, suggest authors add how the ENCODE ChIP-seq data were incorporated into TFutils in the abstract.

**RESPONSE**: metadata(encode690) provides details. The ENCODE data are derived from

Bioconductor's AnnotationHub package. These facts are noted in the vicinity of Figure 4.  We have added a phrase to the abstract that mentions the ENCODE interface.

**QUERY**: Page 5, please clarify the type of details in the sentence “Full details are provided in Sonawane et al”.

**RESPONSE**: These authors describe how FIMO was used to obtain sequence-based binding affinity scores; the main text is slightly modified to clarify the role of this reference.

**QUERY**: Gene structure can be better depicted in Fig. 3 and Fig. 4, perhaps adopting the gene structure visualization in most genome viewers, showing exon/intron structure and gene transcription direction.

**RESPONSE**: We agree that the gene model displays are sub-optimal. However, the visualizations are not central to the package. Ideally, Gviz, ggbio, or karyploteR infrastructure would be used and we will pursue these improvements in updates to the package. Further discussion of the visualization is conducted with the other referee report. We note that the interactive "app" at https://vjcitn.shinyapps.io/encdemo/ can be used to interactively view binding sites for a small number of TFs in a small number of cell types. Such visualizations can be better accomplished with standard genome browsers. The visualizations in the paper are provided to make concrete the immediate programmatic availability of these resources and concepts to package users. In particular, Figure 3 just scratches the surface of the concept that the combinatorics of TF binding are complex. As noted by the other reviewer, the display is impossible to parse meaningfully in detail, but the overall interpretation is that binding events form a complex ensemble and that data structures and programming patterns are needed to develop compelling interpretations.

**QUERY**: Please include the used R packages in the citation.

**RESPONSE**: After running the Rmarkdown document in TFutils/vignette/TFutils_f1000 that conducts all the computations presented in the paper, sessionInfo() can be run to enumerate all packages in use. There are 37 packages attached, and 60 loaded but not attached. It does not seem reasonable to burden the paper with such an accounting. Users can run sessionInfo() and then query the DESCRIPTION files of packages of interest for provenance information.

**QUERY**: “TFtargs()” in Figure 5 legend needs to be edited.

**RESPONSE**: Done.

**QUERY**: For the subtitles under the Use cases section, suggest add find before “TFs that are direct GWAS …” and retrieve before “Traits mapped to genes that …”.

**RESPONSE**: Done.
